# How “moral” are the principles of biomedical ethics? – a cross-domain evaluation of the common morality hypothesis

**DOI:** 10.1186/1472-6939-15-47

**Published:** 2014-06-17

**Authors:** Markus Christen, Christian Ineichen, Carmen Tanner

**Affiliations:** 1 University Research Priority Program Ethics, University of Zurich, Zurich, Switzerland; 2 Institute of Biomedical Ethics, University of Zurich, Zurich, Switzerland; 3 Department of Banking and Finance, University of Zurich, Zurich, Switzerland

**Keywords:** Autonomy, Beneficence, Business and finance, Common morality, Justice, Medicine, Moral psychology, Moral values, Non-maleficence, Principlism

## Abstract

**Background:**

The principles of biomedical ethics – autonomy, non-maleficence, beneficence, and justice – are of paradigmatic importance for framing ethical problems in medicine and for teaching ethics to medical students and professionals. In order to underline this significance, Tom L. Beauchamp and James F. Childress base the principles in the common morality, i.e. they claim that the principles represent basic moral values shared by all persons committed to morality and are thus grounded in human moral psychology. We empirically investigated the relationship of the principles to other moral and non-moral values that provide orientations in medicine. By way of comparison, we performed a similar analysis for the business & finance domain.

**Methods:**

We evaluated the perceived degree of “morality” of 14 values relevant to medicine (n_1_ = 317, students and professionals) and 14 values relevant to business & finance (n_2_ = 247, students and professionals). Ratings were made along four dimensions intended to characterize different aspects of morality.

**Results:**

We found that compared to other values, the principles-related values received lower ratings across several dimensions that characterize morality. By interpreting our finding using a clustering and a network analysis approach, we suggest that the principles can be understood as “bridge values” that are connected both to moral and non-moral aspects of ethical dilemmas in medicine. We also found that the social domain (medicine vs. business & finance) influences the degree of perceived morality of values.

**Conclusions:**

Our results are in conflict with the common morality hypothesis of Beauchamp and Childress, which would imply domain-independent high morality ratings of the principles. Our findings support the suggestions by other scholars that the principles of biomedical ethics serve primarily as instruments in deliberated justifications, but lack grounding in a universal “common morality”. We propose that the specific manner in which the principles are taught and discussed in medicine – namely by referring to conflicts requiring a balancing of principles – may partly explain why the degree of perceived “morality” of the principles is lower compared to other moral values.

## Background

The “*Principles of Biomedical Ethics*” by Tom L. Beauchamp and James F. Childress, which appeared for the first time in 1977, is a classic text in biomedical ethics. The authors’ contribution has been celebrated as one of the most important methodological inventions of modern practical ethics, particularly in Anglophone scholarship [1]. The core features of this so-called principlism are to locate moral principles (autonomy, non-maleficence, beneficence, and justice) pertinent to a particular moral situation and to use specification, balancing and (deductive) application to create a bridge between the moral situation and the relevant principles. In addition, the authors adopt a prescriptive common morality thesis as a theoretical justification for the methodological reasoning within principlism. This grounding of the principles in the common morality was emphasized in later editions. At the beginning of the most recent 7th edition, published in 2013, Beauchamp and Childress state that the common morality “refers to norms about right and wrong human conduct that are so widely shared that they form a stable social compact” ( [2], p. 3). Thus, the common morality is not merely a specific morality, in contrast to other moralities, but is rather applicable to all persons in all places, and we rightly judge all human conduct by its standards. Examples include situations in which, for example, one knows not to lie, not to steal property, to keep promises, to respect the rights of others, not to kill or not to cause harm to innocent persons, and the like ( [2], p. 3; [3]). It has been argued that there is far more consensus on common morality principles and rules than on any other moral theories. Hence, appealing to norms of the common morality will work better for practical decision-making [4].

However, principlism is not undisputed in bioethics. Its virtues are clarity, simplicity and universality, but its vices include neglect of emotional and personal factors that are inherent in specific decision situations, oversimplification of the issues, and excessive claims of universality [5]. The main focus of the critique concerns the completeness of the approach with respect to its practical use for dealing with moral problems in clinical practice given the challenge of ethical pluralism [6]. Opinions are conflicting in this regard. In support of principlism, Gillon [7] argues that the principles enable a clinician (and anybody else) to link professional guidance and rules with ethical aspects. They also allow new situations to be confronted in the light of these acceptable principles. There is some empirical support that the daily work of physicians and other professionals in biomedicine indeed reflects these four principles [8]. Others [9], however, argue that the principlist model is unreflective of how ethical decisions are taken in clinical practice and that the model is neither sufficiently action-guiding nor explicit about how to attain professional integrity. For example, based on an analysis of the communication process with Muslim parents, Westra and colleagues [10] concluded that the parties involved in disagreement may feel committed to seemingly similar, but actually quite different principles. In such cases, communication in terms of the principles may create a conflict within an apparently common conceptual framework. Page [11] found that psychology students value the principles but do not actually seem to use them directly in the decision-making process, which partly calls into question their practical relevance.

Despite such findings, the common morality has been emphasized by Beauchamp and Childress as the ultimate source of moral norms since the 5th edition of the “*Principles of Biomedical Ethics*” [4]. Karlsen and Solbakk [12] observed that with the publication of the 6th edition, the authors not only attempted to ground their theory in the common morality, but that there was also an increased tendency to align the former with the latter. While this strategy may give the impression of a more robust, and hence stable, foundation for the theoretical construct of principlism, Karlsen and Solbakk argue that this comes at the expense of theoretical and practical open-mindedness. In line with this reasoning, Beauchamp and Childress suggested that the question of whether the principles are indeed part of the common morality should be an object of empirical inquiry ( [2], p. 416). Our research seeks to provide a first step of an empirical investigation of the common morality.

### An empirical investigation of the common morality

For the purpose of the empirical investigation, we suggest making use of research in moral psychology. We posit that moral values or principles “so widely shared that they form a stable social compact” ( [2], p. 3) are grounded in psychological processes that allow us to recognize that these values are at stake, and to align decisions and actions with these values. Furthermore, to the extent that these processes and values share some degree of universality, they are built into us by evolution. For example, beneficence or caring for others might be seen as being products of natural selection, adapted to allow survival [13].

In the following, we focus on those findings in moral research that align with the universality claim of the common morality in order to identify “dimensions” of morality that can then be empirically investigated. The findings posit that people have nearly instant reactions to situations of moral violations [14-16], indicating that moral concepts are “chronically accessible” [17] mental representations. This foundation of morality in human psychology developed to solve problems that faced our ancestors for millions of years. For example, generosity and sharing developed due to extreme mutual interdependence [18]. One can therefore expect that the common morality has a rich and long history of heritage across and beyond the hominid line [19], supporting the universality claim. Moreover, cognitive approaches of moral psychology, e.g. the moral-conventional distinction model of Turiel [20], have emphasized universal aspects of morality in the sense that the moral wrongness of an action does not depend on specific circumstances. However, beyond universality, current research in moral psychology and anthropology points out two further dimensions of morality, namely community orientation and cooperation [18]. Thus, it is plausible to relate the common morality, which should be “shared by all persons committed to morality” ( [2], p. 3), to the features universality, community orientation and cooperation.

Certainly, there is also a line of research within moral psychology emphasizing the cultural and social grounding of morality, which calls this universality claim into question and tends to align with the tradition of moral relativism within philosophy [21]. Thus, an empirical investigation of the values that are claimed to be part of the common morality should also include a cross-cultural and/or cross-social comparison.

### Research goals

The aim of the present research is twofold. First, we wish to empirically examine the extent to which various values are considered to be *moral* values and whether this evaluation is characterized by the features of universality, communion and cooperation orientation. With the term ‘value’ , we refer to stable beliefs about desirable states or conducts of behaviors, which serve as normative standards to assess and justify actions [22,23]. Second, we wish to test whether these evaluations of values generalize across different social domains. In doing so, we compare this evaluation in two domains: medicine and business & finance. We hypothesize that morality ratings coincide strongly with the features of universality, communion and cooperation orientation, and that these relations persist across different social domains. From this hypothesis, we deduce that: If the values that relate to the principles are, as expected, commonly characterized by these features, and if these evaluations persist across social domains of application, we have support for the claim that the principles are part of the common morality. If this is not the case, we have a conflicting result that requires further investigation.

For the purpose of our research goals, we began with a qualitative step to identify the values that are considered as relevant in each domain. In the second, quantitative, step, we conducted two domain-specific surveys with the aim of investigating the evaluation of these values.

### Step 1: value identification within two domains

We began by conducting literature reviews, interviews with experts, and a small survey among various professionals in Switzerland to identify a) the relevant values within the respective domain, and b) typical behavioral manifestations of these values. Using this procedure, we identified 14 values considered to be important within the respective domain. These values were not necessarily “genuinely moral”. The values identified in this manner in medicine were: autonomy (Autonomie), care (Fürsorge), cost-effectiveness (Wirtschaftlichkeit), feasibility (indicating that the physician should do whatever is technically possible; technischer Imperativ), honesty (Ehrlichkeit), integrity (Integrität), justice (Gerechtigkeit), loyalty (Loyalität), non-maleficence (Nichtschaden), performance (Leistung), professionalism (Professionalität), reputation (Reputation), respect (Respekt), and responsibility (Verantwortung). The 14 business & finance values were: engagement (Engagement), competition (Wettbewerb), compliance (Regelkonformität), fairness (Fairness), integrity (Integrität), loyalty (Loyalität), non-maleficence (Nichtschaden), performance (Leistung), professionalism (Professionalität), profitability (Profitabilität), reputation (Reputation), respect (Respekt), responsibility (Verantwortung), and transparency (Transparenz). As expected, the values of the two domains only partially overlapped.

To ensure that participants had a precise and common understanding of the values, which was necessary for making an accurate evaluation, each value was presented with three examples. These examples represented typical manifestations of the corresponding value in domain-specific settings and we used domain-specific terms (‘patients’ , ‘customers’, etc.) to adapt the descriptions to the respective domain. For instance, examples for autonomy in medicine were “A person or an institution a) respects the self-determination of others, b) avoids putting pressure on others to reach goals, and c) supports others such that they can make their own decisions.” Examples for profitability in business & finance were “A person or a company a) tries constantly to optimize the relationship between revenue and expenditure, b) defines success as the pursuit of profit, and c) grants a paramount importance to key financial indicators”. Table 1 outlines the value exemplifications used in our study (German original and English translation).

**Table 1:** Value characterization (German original and English translation)

**Domain**	**Value**	**Description German**	**Description English**
		**Eine Person oder eine Institution...**	**A person or an institution…**
M	Autonomie [autonomy]	…achtet die Selbstbestimmung Dritter; …vermeidet die Anwendung von Druck gegenüber Dritten zur Erreichung von Zielen; …unterstützt Dritte, so dass diese eine eigene Entscheidung treffen können	…respects the self-determination of others; …avoids putting pressure on others to reach goals; …supports others such that they can make their own decisions
M	Ehrlichkeit [honesty]	…kommuniziert einem Patienten diagnostische Ergebnisse umfassend; …verschweigt keine negativen Folgen einer Therapie; …informiert die Öffentlichkeit über negative Vorfälle in der eigenen Institution	…communicates diagnostic results to a patient comprehensively; …does not conceal risks of therapies; …informs the public about negative incidents in own institution
B	Engagement [engagement]	…erledigt Aufgaben mit Begeisterung und viel Willenskraft; …scheut keine zusätzlichen Aufwände; …setzt sich für eine Sache ein	…completes tasks with enthusiasm and willpower; …does not shy away from additional efforts…; puts much effort into a task
B	Fairness [fairness]	…beurteilt Bewerbungen unter gleichen Bedingungen; …bevorzugt niemanden bei Beförderungen; …verzichtet darauf, einen Informationsvorsprung auszunützen	…evaluates applications equally; …does not favor someone when it comes to promotion; …refrains from exploiting an information advantage
M	Fürsorge [care]	…hilft Dritten, die in Not sind; …steht für die Bedürfnisse von leidenden Menschen ein; …sorgt sich um das Wohlergehen Dritter	…helps others who are in distress; …protects the interests of people who are suffering; …cares about the welfare of others
M	Gerechtigkeit [justice]	…behandelt Patienten gemäss ihren Bedürfnissen und nicht gemäss ihrem Status; …versucht, verschiedene Standpunkte zu einem Ausgleich zu bringen; …behandelt Mitarbeitende fair	…cares for patients according to their needs and not their social status; …tries to balance different points of view; …treats coworkers fairly
B & M	Integrität [integrity]	…lässt sich nicht korrumpieren; …hält an den eigenen Werten fest; …verdient Vertrauen	…doesn’t allow him/herself to be corrupted; …sticks to own values; …earns trust
B & M	Leistung [performance]	…schätzt Erfolge; …orientiert sich an den Besten; …misst sich über Ergebnisse	…appreciates success; …orients him/herself towards the best; …measures oneself based on results
B & M	Loyalität [loyalty]	…entlässt keine langjährigen Mitarbeiter; …ist treu gegenüber der eigenen Institution; …stellt sich schützend vor die Mitarbeiter	…does not fire long-time employees; …is faithful to own institution; …is protective of his employees
B & M	Nichtschaden [non-malefience]	…unterlässt riskante medizinische Interventionen bei zweifelhaften Erfolgsaussichten; …vermeidet es, bei einer Therapie einen Patienten zu schädigen; …minimiert das Leiden eines Patienten.	…refrains from risky interventions with dubious prospects of success; …avoids harming a patient during a treatment; …minimizes a patient’s suffering
B & M	Professionalität [professionalism]	…bemüht sich aktiv um die Einhaltung geltender Gesetze und Standesregeln; …prüft Patientenanfragen sorgfältig und gewissenhaft; …erledigt Aufgaben gründlich	…strives for compliance with laws and rules of professional conduct; …checks patient inquiries carefully and scrupulously; …completes tasks thoroughly
B	Profitabilität [profitability]	…versucht stets, das Verhältnis von Einnahmen und Ausgaben zu optimieren; …beurteilt Erfolg als Erzielen von Gewinn; …misst finanziellen Kennzahlen eine hohe Bedeutung zu	… tries constantly to optimize the relationship between revenue and expenditure; … defines success as the pursuit of profit; … grants a paramount importance to key financial indicators
B	Regelkonformität [compliance]	…befasst sich mit internen Verhaltensmassstäben; …prüft die Einhaltung von internen Regelungen; …hält sich an offizielle Vorschriften.	…is concerned with codes of conduct; …checks the compliance with internal rules; …adheres to official regulations
B & M	Reputation [reputation]	…orientiert ihr/sein Handeln an der Meinung anderer; …achtet darauf, einen guten Ruf zu wahren; …ist eine anerkannte Kapazität auf ihrem Gebiet	…orients his/her behavior to the opinion of others; …tries to maintain a good reputation; …is a recognized authority in his/her field of expertise
B & M	Respekt [respect]	…akzeptiert individuelle Unterschiede von Personen; …begegnet Patienten hochachtungsvoll; …respektiert die Privatsphäre von andern.	…accepts people’s individual differences; …encounters patients respectfully; …honors others’ privacy
M	Technischer Imperativ [feasibility]	…setzt alle möglichen Mittel für Therapie und Pflege ein; …sieht die Bewahrung von Leben als oberstes Gebot an; …rettet Menschen unter allen Umständen	…uses all possible means for treatment and care; …considers the saving of life as the highest priority of all; …saves persons under any circumstances
B	Transparenz [transparency]	…kommuniziert offen und ehrlich über anstehende Veränderungen; …macht neue Entwicklungen nachvollziehbar für Dritte; …informiert über Risiken, die aus ihren Tätigkeiten entstehen können	…communicates openly and honestly about upcoming changes; …makes new developments comprehensible for others; …informs about risks that could result from own actions
B & M	Verantwortung [responsibility]	…steht für negative Folgen seiner Tätigkeiten gerade; …berücksichtigt Ansprüche der Gesellschaft; …lagert Risiken nicht aus und nimmt seine Pflichten wahr	…is answerable for negative consequences of own actions; …considers demands of society; …does not shift risks onto others, and takes his duties seriously.
B	Wettbewerb [competition]	…steht im Wettstreit mit anderen und möchte sich durchsetzen; …mag Konkurrenzsituationen; …möchte erfolgreicher sein als andere.	…competes with others and wants to assert him/herself; …likes rivalry; …wants to be more successful than others
M	Wirtschaftlichkeit [cost-effectiveness]	…beachtet die Kostenfolgen von Therapieentscheidungen; …ist sich bewusst, dass für das Gesundheitswesen keine unbegrenzten Mittel eingesetzt werden können; …setzt Mittel kosteneffizient ein.	…is concerned about the costs of treatments; …is aware that the available financial resources for the health system are limited; …uses means in a cost-efficient way

B: Business & finance, M: Medicine.

The wording of descriptions of values that appear in both domains can be adapted slightly (e.g. “customer” instead of “patient”); in the table, the descriptions used in the medical domain are shown.

We note that the term ‘care’ (Fürsorge) and not ‘beneficence’ was used in the survey, since the German ‘Fürsorge’ is more common than the technical term ‘Benefizienz’. Furthermore, we used general descriptions of ‘fairness’ and ‘transparency’ for business & finance that were similar to the descriptions of ‘justice’ and ‘honesty’ in medicine. In other words, the semantics of these terms overlap, although there is a domain-specific tradition that, for example, what is considered a matter of honesty (Ehrlichkeit) in medicine is often a matter of transparency (Transparenz) in business & finance. Thus, overall, we had eight values that are commonly present in both domains and two domain-specific values that shared a large degree of semantic similarity. As expected, for the medicine domain, the experts considered all four principles to be important values; for the business & finance domain, two out of the four principles (non-maleficence and justice/fairness) were considered as relevant.

### Step 2: value evaluations within domains

The following investigation was designed to examine the evaluations of the values identified in the previous step by conducting two domain-specific surveys.

## Methods

We collected data from two samples through online surveys. In total, 455 participants composed of students and staff of the University of Zurich (the focus was on medical students, but students of other faculties could also participate) and members of a network of health professionals provided data for the medicine survey. Regarding the business & finance survey, the sample consisted of 333 economics students and staff (most of whom had work experience in business or finance). This study was cleared in accordance with the ethical review processes of the University of Zurich and within the “Ethical Guidelines for Psychologists of the Swiss Society for Psychology” (http://www.ssp-sgp.ch/06_pdf/ersgp2003.pdf). Furthermore, we followed the CHERRIES guidelines (The Checklist for Reporting Results of Internet E-Surveys; see http://www.jmir.org/2004/3/e34/) insofar as they were applicable to the surveys.

Each survey contained two parts. After participants had provided informed consent, we first assessed demographic information (gender, age, field of study) and information about the participants’ work experience in medicine or business & finance (whether they had work experience, and what kind of experience). Participants then rated each value (in a randomized order) along four dimensions using a 6-point Likert scale (see below). The participants were able to quit the survey whenever they wished. The participants who completed the whole survey were entered into a lottery to win an iPad. After quitting or after having rated all 14 values, the participants were asked whether they completed the survey with due diligence (this is a standard test question in psychological online surveys, but the answer did not influence whether the participants were entered into the lottery). Those participants who stated that they did not complete the survey with due diligence or did not answer the question were excluded from the data sets. We also compared the time needed to complete the survey between participants negating or affirming the due diligence question. Assuming that individuals who completed the survey with less care would need less time, we also excluded participants who responded as quickly as those who stated that they did not complete the survey with due diligence (this was only the case for participants who had only rated one single value).

After this quality check, the samples consisted of 317 participants in medicine (dropout: 30.3%) and 247 participants in business & finance (dropout: 25.8%). In the medicine sample, 71.9% of the participants were female, their mean age was *M* = 25.4 years, and 27.4% reported having work experience (24.9% provided detailed descriptions of their profession, e.g. working as physician or nurse). In the business & finance sample, 54.3% of the participants were female, their mean age was *M* = 26.6 years, and 31.6% reported having work experience (21.9% reported that they currently work between 50-100% in the business or finance domain). The over-representation of females in the medicine survey is mainly explained by the fact that 61% of medical students in Switzerland are female (final degree, data of 2010; [24]) and that among the professionals, more nurses than doctors answered the survey.

In the main part of the survey, we assessed the participants’ evaluation of the corresponding value along four dimensions (see Table 2): The “moral – non-moral” dimension was explicitly described as referring to universal principles and issues of right and wrong (MO-NMO) [20]. The “community-oriented – self-oriented” dimension referred to the social notion of morality (COM-SELF) [25]. The “cooperative – competitive” dimension was described as referring to collaborative or rivalry tendencies between human beings or institutions (COOP-COMP) [18]. Finally, we added the “principle-focused – consequentialist” dimension in order to include a reference to the classic teleological vs. deontological distinction in ethical theory (PRI-CON). This also served as a test to examine whether the notions of autonomy, care (beneficence), non-maleficence and justice are actually evaluated as “principles” as in the approach of biomedical ethics; we found no indications in this regard. Each dimension was rated on a 6-point scale (1 = *moral; community-oriented, cooperative,* or *principle-focused*; 6 = *non-moral, self-oriented, competitive,* or *consequentialist*).

**Table 2:** Dimensions characterizing morality and their description in the survey (English translation)

**Dimension**	**Description of left endpoint**	**Description of right endpoint**
MO-NMO: moral – non-moral	A value is “moral” if it claims to be universally valid and its corresponding actions are judged as right or wrong.	A value is “non-moral” if it is not claimed to be universally valid and if corresponding actions are not judged as right or wrong.
COM-SELF: community-oriented – self-oriented	A value is “community-oriented” if it refers to the goals of a community, common interest or the relationships among individuals.	A value is “self-oriented” if it refers to the priority of personal goals, personal interests or the individual.
COOP-COMP: cooperative – competitive	A value is “cooperative” if it refers to the collaboration, cooperation or communication between human beings or institutions.	A value is “competitive” if it refers to the competition or rivalry between human beings or institutions.
PRI-CON: principle-focused – consequentialist	A value is “principle-focused” if it focuses on the legitimacy of the act itself when the value is used to evaluate actions.	A value is “consequentialist” if it focuses on the evaluation of consequences of an action when the value is used to evaluate actions.

## Results

We will first report the bivariate correlations among the dimensions, followed by the evaluation of single values across domains. Finally, we will examine how the values based on similarity analyses cluster within each domain. The data were analyzed using the software package Mathematica® version 9.

### Correlational analyses

For each domain, we examined the pairwise Pearson product–moment correlations among the four dimensions at the aggregated level (across all values) and at the specific level (for each single value). Table 3 reports both the findings with the aggregated data, and the number of values with significant (*ps* < 0.05) bivariate correlations. As can be seen from Table 3, the mutual correlations among dimensions MO-NMO, COM-SELF and COOP-COMP are about twice as high as the correlations among MO-NMO, COM-SELF, COOP-COMP and PRI-CON. At the aggregated level: The mean correlations among the first three dimensions in medicine and business & finance are 0.52 and 0.61, while the mean correlations of these with the fourth dimension are 0.25 and 0.34. A similar picture emerges at the specific level: The mean number of values with significant correlations among the first three dimensions in medicine and business & finance are 12.3 and 12.7, while the mean number of values with significant correlations with the fourth dimension are 5.0 and 5.0. These results demonstrate that the dimensions MO-NMO, COM-SELF and COOP-COMP are more closely associated among themselves than with dimension PRI-CON. We thus conclude that participants tend to associate a “moral value” with the attributions: universally valid, an issue of right and wrong, community and cooperation. In contrast, a “non-moral value” tends to be characterized by the features: non-universal, not an issue of right and wrong, but an issue of self-orientation and competition. Note that this “moral” versus “non-moral” distinction correlated only weakly with the “principle-focused” versus “consequentialist” distinction. One reason might be that, referring to the classic ethical traditions, a moral value can imply either a deontological or a consequentialist focus. Hence, weaker correlations are likely. We thus restrict the following cluster analysis to the dimensions MO-NMO, COM-SELF and COOP-COMP.

**Table 3:** Pearson product–moment correlation among the four dimensions (*p < 0.05, ***p < 0.001)

**Correlated dimensions**	**Medicine (*n1*** **= 317)**		**Business & finance (*n2*** **= 274)**	
	**Correlation of aggregated data**	# of values with significant (*) correlation	**Correlation of aggregated data**	# of values with significant (*) correlation
MO-NMO with COM-SELF	0.41***	10	0.53***	10
MO-NMO with COOP-COMP	0.58***	13	0.63***	14
COM-SELF with COOP-COMP	0.58***	14	0.68***	14
MO-NMO with PRI-CON	0.29***	7	0.35***	4
COM-SELF with PRI-CON	0.20***	5	0.31***	5
COOP-COMP with PRI-CON	0.24***	3	0.37***	6

### Evaluation of single values

Table 4 compares the ratings for the single values across medicine and business & finance (only for the eight values that were present in both domains). As can be seen, the analyses revealed significant differences with regard to five values. When contrasting medicine with business & finance, loyalty is less moral (dimension MO-NMO); responsibility is more self-centered (COM-SELF); and performance is more competitive (COOP-COMP) in medicine. Reputation and non-maleficence are less “moral” in medicine across all three dimensions.

**Table 4:** Comparing dimension MO-NMO, COM-SELF and COOP-COMP ratings of values across medicine and business & finance (*p < 0.05, **p < 0.01, ***p < 0.001)

	**MO-NMO**	**COM-SELF**	**COOP-COMP**
Respect	n.s.	n.s.	n.s.
Loyalty	0.29*	n.s.	n.s.
Responsibility	n.s.	0.33*	n.s.
Reputation	0.49**	0.51***	0.69***
Performance	n.s.	n.s.	0.38**
Professionalism	n.s.	n.s.	n.s.
Non-maleficence	0.52***	0.84***	0.37***
Integrity	n.s.	n.s.	n.s.

Further analyses also revealed differences when contrasting participants with work experience to students without work experience, both in medicine and business & finance (data not shown; Mann–Whitney test, *ps* < 0.05). Participants with professional experience in medicine consider loyalty to be significantly less moral (dimension MO-NMO) and justice to be significantly more cooperative (COOP-COMP). Participants with professional experience in business & finance consider engagement to be significantly more community-oriented (COM-SELF), reputation to be more non-moral (MO-NMO), self-centered (COM-SELF) and competitive (COOP-COMP), and integrity to be more cooperative (COOP-COMP).

### Value classification

**Figure 1:**
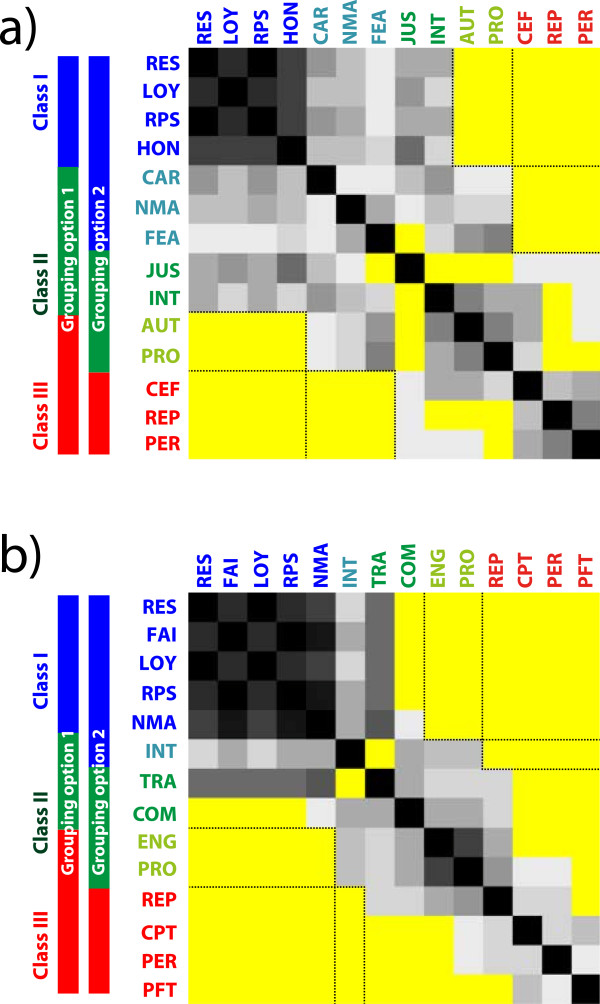
**Count matrix representing how often two values have been classified in the same group: the darker the entry, the more often two values have been grouped together (maximum 12 times).** Yellow entries indicate values that have never been classified together; **a)** count matrix for medicine, **b)** count matrix for business & finance. The color bars on the left side indicate the two grouping options (blue: class-I, green: class-II, red: class-III). Value abbreviations: AUT = autonomy; CAR = care, CPT = competition, COM = compliance, CEF = cost-effectiveness, ENG = engagement, FAI = fairness, FEA = feasibility, HON = honesty, INT = integrity, JUS = justice, LOY = loyalty, NMA = non-maleficence, PER = performance, PRO = professionalism, PFT = profitability, REP = reputation, RES = respect, RPS = responsibility, TRA = transparency.

In a next step, we analyzed the classification of the values using two similarity metrics and two classification methods for each dimension MO-NMO, COM-SELF and COOP-COMP separately. For the similarity metrics, we used Mann–Whitney and Kolmogorov-Smirnov as two complementary nonparametric tests (the former has a higher power for rejecting the null hypothesis, the latter is more sensitive to the form of the distribution, e.g., bimodality). In the first classification method, two values are considered to be in the same group if the ratings along one dimension are not distinguishable for one of the two tests (i.e., *ps* > 0.05). In the second classification method, the p-values of the two tests were used to create a distance matrix for either test (each matrix element is calculated as 1 minus the p-value of the corresponding value pair). Either the MW or the KS distance matrix for one dimension then served as input for a clustering algorithm that required no predefined specifications on cluster number and size [26]. In this way, two values X and Y could be in the same group a maximum of 12 times (3 dimensions × 2 similarity measures × 2 classification methods).This, in turn, resulted in a count matrix in which each matrix element stands for the number of times the two associated values have been put in the same group (Figure 1). The matrix rows can be ordered such that those values that are frequently grouped together are neighbors. Finally, these analyses revealed three classes of values with the following features: class-I (blue) and class-III (red) values are completely distinct; i.e. values from class I were never grouped together with values from class III or vice versa. In contrast, class-II (dark green) values tend to overlap with the other two classes, i.e. for some combination of dimension, similarity measure and classification method, a class-II value is grouped with a class-I value, and for some other combination, it is grouped with a class-III value.

A closer analysis of the count matrix reveals two ways of forming these three value groups. Note that class I (blue) and class III (red) encompass the moral and non-moral group, respectively. In medicine, and with respect to grouping option 1, the blue class is composed of the values respect, loyalty, responsibility and honesty, while autonomy joins the red class. With respect to grouping option 2, the blue class is extended by the values care, non-maleficence and feasibility, while the red class is composed of the values cost-effectiveness, reputation, and performance. In business & finance, similar findings are discernible: In grouping option 1, the blue class is composed of the values respect, fairness, loyalty, responsibility, and non-maleficence, while the red class includes the values engagement and professionalism. In grouping option 2, the blue class is extended by the value integrity, while engagement and professionalism are excluded from the red class.

Taking the intersection of these two approaches of analyses reveals a “moral” core and a “non-moral” core for both social domains, which is partially domain-overlapping and partially domain-specific. In medicine, the moral core consists of respect, loyalty, responsibility and honesty; in business & finance, it consists of respect, fairness, loyalty, responsibility and non-maleficence. These values have been classified together in almost all cases. In medicine, the non-moral core consists of cost-effectiveness, reputation and performance; in business & finance, it is composed of the values reputation, competition, performance and profitability. Note that both the moral and non-moral cores share a high degree of overlap between the medicine and business & finance domain: respect, loyalty and responsibility for the moral core, and reputation and performance for the non-moral core.

**Figure 2:**
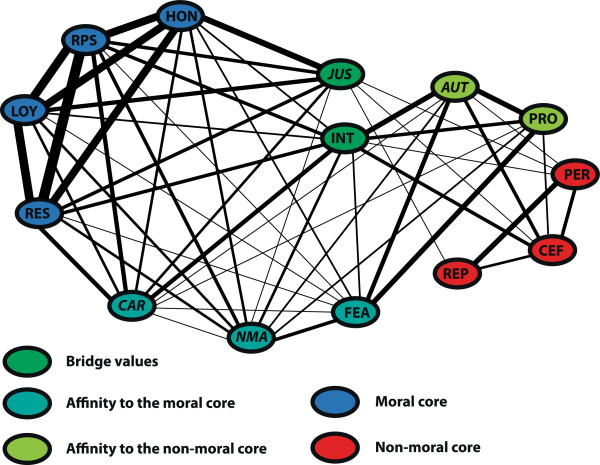
**Network representation of the count matrix in medicine.** The size of the edge between two values represents how often these values have been grouped together. Value abbreviations: see caption Figure 1.

**Figure 3:**
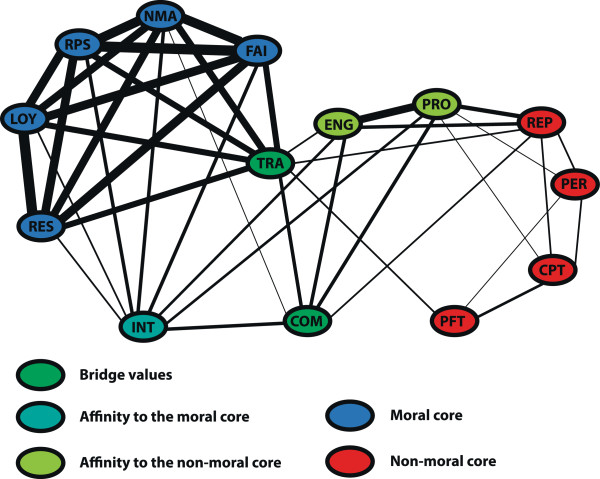
**Network representation of the count matrix in business & finance.** The size of the edge between two values represents how often these values have been grouped together. Value abbreviations: see caption Figure 1.

Strikingly, none of the four values related to the principles of biomedical ethics (autonomy, non-maleficence, care, and justice) fall into the moral core; autonomy even received consistently low ratings among all three dimensions, making it almost a class-III value. In contrast, in the business & finance domain, two of the four values that represent the principles – non-maleficence and fairness – are in the moral core.The result of the count matrix can also be displayed as a network, with the size of the edges between two values reflecting the frequency with which the corresponding two values have been classified together (Figures 2 and 3). This representation motivates the notion of class-II values as “bridge values” (marked in green), i.e. these values can be grouped either with moral or with non-moral values depending on dimension, similarity metrics and classification method. Some “bridge values” have a stronger affinity to the moral core (marked in turquoise: care, feasibility and non-maleficence in medicine; integrity in business & finance), whereas others have a stronger affinity to the non-moral core (marked in green: autonomy and professionalism in medicine; engagement and professionalism in business & finance).

## Discussion

We hypothesized that we can characterize the common morality using three dimensions which have been derived from current empirical research in morality. As expected, we found that these dimensions correlate strongly across the social domains medicine and business & finance. In addition, we identified values that form a moral core within both domains – respect, loyalty and responsibility. These data are consistent with the notion of a common morality, i.e. there are values that are perceived as being highly moral across social domains.

Strikingly, we found that the values associated with the principles of biomedical ethics are not part of this moral core. In particular, based on the ratings given by the participants, it is questionable whether non-maleficence and in particular autonomy are perceived as being part of the common morality. Interestingly, in the business & finance domain, non-maleficence is part of the moral core, indicating a domain-specificity of the perceived morality of this value. These findings are in conflict with the common morality hypothesis of Beauchamp and Childress. However, they are in line, for example, with Haidt and Joseph [19], who propose that innately prepared intuitions generate social-culturally variable values and virtues.

From the point of view of medical ethics, at first glance, our finding may be surprising, if not worrying, because one may consider it to be an indication of a failure to convey the desired normativity of values to the professionals who should work with them. Furthermore, the finding might indicate that the principles – in particular non-maleficence and autonomy – may not be grounded in the moral psychology of medical professionals in the same way as other moral values. This raises the question of how principles which are inherently not as moral-laden as assumed guide health care providers in conflict situations to find a helpful – and for their part “moral” – orientation that would generate guidance. We believe that to answer this question, one should analyze the function of the principles in practical moral decision making. Page [11] found an absence of predictive power of the principles in decision making, and concluded that this may be due to the absence of a behavioral model explaining how individuals cognitively use these principles in their decision making. According to our model of moral intelligence, which is proposed to provide an integrative framework for understanding moral behavior [27], moral values are, if internalized, part of the individual’s “moral compass” that helps to guide behavior. The weaker this grounding is – and lower “morality ratings” indicate this – the less likely it is that decision problems are framed as moral problems and that the corresponding values come into play in the decision-making process as moral values.

We furthermore suggest that the way in which the principles are discussed and learned within biomedical ethics – namely as instruments to deal with dilemmatic situations – influences to some degree their grounding in the individual’s moral psychology. If values like non-maleficence or autonomy are regularly discussed in cases that involve a conflict between them, it is likely that the initial appeal of understanding autonomy as “moral” (i.e., providing unambiguous action guidance) is weakened. In this respect, it is interesting that professionals in our survey consider, for example, loyalty to be significantly less moral (dimension MO-NMO) compared to the students, although loyalty is considered to be among the moral foundations whose moral psychology has an evolutionary history [19]. As the moral complexity of many clinical problems can often be understood as conflicts in loyalties (e.g. between head physician and patient), it is possible that these experiences weaken the initial moral appeal of loyalty. That is, the social practice of dealing with the principles in a specific way in biomedical ethics (e.g., as instruments to teach ethics to students and health professionals) may at least to some degree foster or erode the foundation of the principles in common morality.

A prediction from our findings is, for example, that – compared to violations of honesty or respect – medical professionals will be less likely to quickly identify violations of autonomy in specific practical clinical problems as a moral issue (due to the particularly low morality ratings of autonomy). The multiple ways of understanding autonomy in medical decision problems [28] make such a prediction plausible.

In addition, our finding is in accordance with some of the recent critique of principlism raised by other scholars. For example, Lee [1] discussed the principles using the distinction between thin and thick concepts, where ‘thin’ and ‘thick’ both have two different meanings: One possibility is to view the terms based on their theoretical status: An ethical theory, method or principle is ‘thin’ in that it covers a theoretical area of morality but thick in that it provides guidance in practical moral realms. The other possibility is to view the terms from the standpoint of content: For example, a theory, method or principle is thin in that it deals with particular moral norms/virtues in a minimal sense but thick in that it utilizes a large number of moral norms/virtues including their cultural or traditional imprinting. Of course, these two ways of using thin and thick overlap in many instances. In the case of principlism, the method is, according to Lee, thick in status, since the method deals with practical moral issues, but thin in content, because it allows different individuals and cultures/traditions to use the four principles in their own way. In that sense, principlism would properly work primarily within Western culture.

In actual fact, our findings indicate an even stronger undermining of the psychological grounding of the principles in the common morality, because even within the same cultural frame we find that the degree of perceived morality of a value differs between social domains. The fact that non-maleficence is unambiguously in the moral values class in the domain business & finance may indicate that the participants in our study tended to frame harming a client or business partner as morally bad, whereas in the medical context, harming a patient is seen as morally justifiable in some contexts (e.g. in the case of vaccination or surgery; note that medical interventions are an incidence of bodily injury from a legal point of view). Thus, the professional training of medics requires them to question the primarily moral appeal of non-maleficence.

Several shortcomings of our study should be noted: First, we cannot completely rule out that the values varied in their generality. That is, the fact that our participants gave higher morality ratings to respect, loyalty, honesty and responsibility over more specific bioethical principles may simply reflect that some of the former values may already include some of the more specific principles. For example, Beauchamp and Childress call their first principle ‘respect for autonomy’ , and the notion of ‘responsibility’ may already involve a ‘duty of care’. Nonetheless, if a more specific value is inherently related to a more general one, we would expect this to also be reflected empirically in a higher similarity between these values regarding the various morality dimensions. In line with this, our results revealed, for example, that ‘responsibility’ is quite strongly associated with care, but that ‘respect’ is not at all associated with ‘autonomy’ (see Figure 2). A reason for this could be that the notion of “respecting a value” can be used for any value term, i.e. the meaning of “respecting x” is different from the general understanding of the value ‘respect’. Furthermore, the argument that in our study, some values were more fundamental that others cannot explain why there are still, and also domain-specific, differences between, for instance, loyalty, non-maleficence and justice/fairness – values for which there are good arguments [19] that they are generic. Of course, however, further research is needed to test the replicability and robustness of our findings.

Second, our approach did not include an intercultural comparison, which would allow for a more valid analysis of the common morality hypothesis. This could be an additional promising extension of this study, although it needs to be taken into account that the translations of value descriptions into different languages would have to be carefully validated in order to avoid shifts in meaning.

## Conclusions

Based on our findings, we can conclude that the principles of biomedical ethics – in particular autonomy – have only a weak grounding in human moral psychology and thus in the common morality. Compared to other moral values, the principles do not appear to be as “inbuilt” or internalized values as expected. This might be unproblematic when people are able to engage in decision-making processes that involve effortful and reflective thinking. In such situations, the principles of biomedical ethics may serve as a useful framework and means for deliberate moral justifications [29]. Research has shown that expenditure of cognitive effort is, for example, more likely under conditions of opportunity (such as low time pressure or low mental workload). However, under conditions of lack of opportunity (such as high time pressure or high mental workload), individuals are more likely to rely on spontaneous processing, and therefore on values that are more internalized and quickly accessible [30]. In such situations, and to the extent that the principles of biomedical ethics are inbuilt in the human mind, they are less likely to affect decision making and behavior. Of course, future studies will have to examine more thoroughly the extent to which the famous biomedical principles really do influence moral decision making and behavior in practical contexts.

## Competing interests

The authors declare that they have no competing interest.

## Authors’ contributions

MC and CT designed the study, MC and CI executed the study, MC performed the data analysis. All authors were involved in preparing the manuscript. All authors read and approved the final manuscript.

## Authors’ information

MC is a researcher in neuroethics and empirical ethics, and research network manager at the University Research Priority Program Ethics of the University of Zurich. CI is a PhD student at the Institute of Biomedical Ethics of the University of Zurich and works in neuroethics. CT is a psychologist and conducts research in ethical decision making. She is currently head of the Center for Responsibility in Finance at the Department of Banking and Finance, University of Zurich, Switzerland.

## Pre-publication history

The pre-publication history for this paper can be accessed here:

http://www.biomedcentral.com/1472-6939/15/47/prepub
